# The movement ecology of seagrasses

**DOI:** 10.1098/rspb.2014.0878

**Published:** 2014-11-22

**Authors:** Kathryn McMahon, Kor-jent van Dijk, Leonardo Ruiz-Montoya, Gary A. Kendrick, Siegfried L. Krauss, Michelle Waycott, Jennifer Verduin, Ryan Lowe, John Statton, Eloise Brown, Carlos Duarte

**Affiliations:** 1 School of Natural Sciences and Centre for Marine Ecosystems Research, Edith Cowan University, Joondalup, 6027 Western Australia, Australia; 2 School of Earth and Environmental Sciences, Australian Centre for Evolutionary Biology and Biodiversity, The University of Adelaide, Adelaide, 5001 South Australia, Australia; 3 The University of Western Australia Oceans Institute, Crawley, 6009 Western Australia, Australia; 4 School of Plant Biology, The University of Western Australia, Crawley, 6009 Western Australia, Australia; 5 School of Earth and Environment, The University of Western Australia, Crawley, 6009 Western Australia, Australia; 6 School of Environmental Systems and Engineering, The University of Western Australia, Crawley, 6009 Western Australia, Australia; 7 Kings Park and Botanic Garden, Botanic Gardens and Parks Authority, West Perth, 6005 Western Australia, Australia; 8 Murdoch University, Murdoch, 6150 Western Australia, Australia; 9 Mediterranean Institute for Advanced Studies, IMEDA (UIB-CSIC), 07190 Esporles, Islas Baleares, Spain

**Keywords:** dispersal, pollen, seed, clonal, marine

## Abstract

A movement ecology framework is applied to enhance our understanding of the causes, mechanisms and consequences of movement in seagrasses: marine, clonal, flowering plants. Four life-history stages of seagrasses can move: pollen, sexual propagules, vegetative fragments and the spread of individuals through clonal growth. Movement occurs on the water surface, in the water column, on or in the sediment, via animal vectors and through spreading clones. A capacity for long-distance dispersal and demographic connectivity over multiple timeframes is the novel feature of the movement ecology of seagrasses with significant evolutionary and ecological consequences. The space–time movement footprint of different life-history stages varies. For example, the distance moved by reproductive propagules and vegetative expansion via clonal growth is similar, but the timescales range exponentially, from hours to months or centuries to millennia, respectively. Consequently, environmental factors and key traits that interact to influence movement also operate on vastly different spatial and temporal scales. Six key future research areas have been identified.

## 1. Movement ecology paradigm

The movement of organisms has profound influence on population, community and ecosystem dynamics over contemporary and evolutionary timescales. Multiple areas of research have focused on the internal and external stimuli that lead to movement, and the consequences of this movement. For plants, unlike most animals, movement is limited to particular life-history stages, i.e. the dispersal of pollen or seed, although long-living clonal plants can grow and hence move slowly over large distances. This review applies a movement ecology framework to enhance our understanding of the causes, mechanisms and consequences of movement in highly clonal, marine angiosperms, the seagrasses.

A general movement ecology paradigm was recently proposed that integrated conceptual, theoretical, methodological and empirical frameworks for studying the movement of organisms [1]. This paradigm developed a common language and identified a unified research agenda on movement. It has been specifically applied to plants by addressing the obvious dichotomy between evolutionary and ecological elements that occur in plants [2]. Conceptually, this model defines the movement path of an individual as a function of how (*Ω*—motion capacity), where (*Φ*—navigation capacity) and why it moves (*W*—internal state), and how these interact with external factors (*R*) [1]. The plant-centric model categorizes external factors into abiotic and biotic dispersal vectors and environmental state [2]. Unlike animals, evolutionary rather than ecological timescales influence motion capacity, internal state and with some exceptions, navigation capacity of plants, but external factors are influenced by ecological timescales [2]. Although the application of this paradigm to plants is increasing, most movement studies (80%) have been on mobile fauna [3]. Relative to terrestrial systems, there is less research in marine systems, where a unique set of abiotic and biotic factors influence movement.

The primary challenge in applying the movement ecology framework to a particular system is identifying the key external factors (*R*), internal states (*W*), motion (*Ω*) and navigation capacities (*Φ*) [1,2]. This review examines the significance and mechanisms of movement in seagrasses by assessing these components. We identify key external factors (biotic and abiotic dispersal vectors, environment) as well as internal states and navigation capacity, which influence the movement path. Then, we assess the motion capacity of four key life-history stages: pollen, sexual propagules (non-buoyant and buoyant seeds and fruits, viviparous seedlings, specialized shoots, e.g. spathes, cymes or rhipidia), vegetative fragments and clonal spread of adult plants. Case studies from selected genera are presented to demonstrate movement paths over space and time. The role and consequences of movement in seagrasses over ecological and evolutionary timeframes will be improved and knowledge gaps identified.

## 2. Seagrasses

Seagrasses are foundation species that inhabit shallow waters of most of the world's coasts and contribute substantially to ecosystem services [4]. They are a polyphyletic group of monocotyledons, order Alismatales that reinvaded the marine environment around 80 Ma [5,6]. There are high frequencies of dioecy among species and hydrophilous pollination, uncommon in flowering plants [7]. In addition, they tolerate a saline medium, grow submerged, have an anchoring system to withstand tidal and wave action and can disperse in the marine environment [8]. Seagrasses are under increasing pressure from human activities and globally are declining at a rapid rate [9]. Understanding the movement ecology of seagrasses provides a way to assess the capacity of populations to recover from impacts associated with existing and future pressures. These include the (re)-colonization of altered or fragmented landscapes, and movement associated with climate change. This information is critical to our understanding of the role of movement on their ecology, evolution and conservation.

## 3. External ecological factors (*R*) impacting movement: dispersal vectors

The marine environment acts as an abiotic dispersal vector and its physical properties significantly influence movement, presenting both challenges and opportunities that differ from terrestrial environments. Typical flow speeds in the ocean are around 0.1 m s^−1^, generally one to two orders of magnitude weaker than typical atmospheric flows (1–10 m s^−1^), that can limit dispersal [10]. However, as seawater density is approximately 1000 times greater than air, momentum of a moving mass of water at the same speed is three orders of magnitude greater than in air. Therefore, drag forces acting on individuals (proportional to density) are also three orders of magnitude higher, enabling relatively larger-sized propagules to be mobilized. But most importantly, buoyancy forces (proportional to the density difference between seawater and the propagule) significantly reduce the effective weight of submerged propagules [11]. Within seagrasses, propagules can weakly settle (negatively buoyant), remain effectively suspended in the interior of the water column (neutrally buoyant), or float at the surface (positively buoyant [12]). With positive buoyancy (e.g. floating fruit), ocean surface currents freely move propagules, and dispersal distances are only limited by the viability time of the fruit [13,14], leading to exceptionally long single dispersal events (more than 100 km, [15]), which is rare for passive abiotic movement of terrestrial fruit and seeds [16]. We identify three motion capacities associated with water motion.

There are a variety of biotic vectors for seagrasses, as they feed on, or live in, seagrass habitat. These include dugongs, manatees, turtles, waterfowl, fish and invertebrates [17–20]. Each biotic vector has its own internal state, motion capacity, navigation capacity and external factors influencing its movement. These interact with plant movement ecology to determine the ultimate movement path of the plant [2,21]. For example, a waterbird will feed on seagrass, if the seagrass contains fruit, and the seed viable after defecation, the bird has the potential to transport seeds from one feeding ground to another. Therefore, the movement path of the bird determines the potential movement path of the seed. Particular traits of the animal, e.g. digestive passage time, directly influence the plant's movement path and likewise for plants, e.g. viability after defecation and viability time. These interact to affect the plant movement path and can be considered a motion capacity (discussed below).

## 4. External ecological factors (*R*) impacting movement: the environment

The state of the environment encompasses all other environmental conditions that interact with the navigation capacity, motion capacity and internal state of the plant, as well as the dispersal vectors, to influence movement. A key environmental state is the landscape, which has a significant impact on the movement of individuals and the ecological, genetic and evolutionary consequences [22,23]. Environmental barriers can significantly impact movement, and in the marine realm, these include factors such as depth, substrate, temperature and salinity. Human constructions, i.e. marinas and changes in sea-level can create barriers, particularly when the connectivity of coastal lagoon systems is impacted. The relevant spatial-scale of these landscape features varies depending on the life-history stage [24]. For pollen, the important landscape features would be canopy height or density and the spatial arrangement of the flowers [25]. For seeds, features such as the distribution and size of seagrass patches and meadows will determine the availability of sites for recruitment [26]. As for all clonal plants, the distribution and size of genets within meadows affects the distance between male and female plants (for dioecious species) and impacts on selfing via geitonogamy. For self-incompatible species, geitonogamy impacts pollen movement, seed set and ultimately the potential for dispersal by seed [27,28]. Finally, environmental conditions such as temperature, aerial exposure, tidal height, wave energy or nutrients can also influence movement [25,29–35]. These are discussed in more detail in §6.

## 5. Evolutionary processes impacting movement: navigation capacity (*Φ*) and the internal state (*W*)

Within the framework of plant movement ecology, the internal state, motion capacity, and in most cases, navigation capacity are driven by traits evolved over multi-generational timescales [2,21]. Like most terrestrial flowering plants, seagrasses have limited navigation capacity [2]. Physiological mechanisms that target the timing of release of seeds to particular sets of environmental conditions to enhance the probability of long-distance dispersal provide some capacity for navigation [16] (electronic supplementary material, S1). There are also examples of navigation capacity over ecological timescales where individuals direct growth to avoid or seek out particular environmental conditions, such as nutrients [36].

Each life-history stage has a specific internal state, or motivation for movement. The internal state is difficult to apply from a plant perspective, owing to the evolutionary timescales over which it operates [21]. Here, we identify the evolutionary impetus for movement. For pollen, the internal state is to find a receptive stigma and achieve fertilization. For reproductive propagules (seeds), it is to find a suitable location for germination and establishment. For clonal growth, it is to persist, increase in size, access resources and enable interaction with other genets. For vegetative fragments, it is to facilitate dispersal and establishment in a suitable location for new growth. Therefore, for movement to be considered successful, pollination, seedling recruitment and vegetative fragment recruitment must occur. The navigation capacity, internal states and external factors, both biotic (e.g. animals) and abiotic (e.g. hydrodynamics) dispersal vectors, as well as the state of the environment (e.g. canopy structure, patchiness, population structure, light, temperature, nutrients) all interact to influence the movement path. Specific plant traits that interact with the external environment to affect movement are presented within the section on motion capacity.

## 6. Motion capacity (*Ω*)

**Figure 1.**
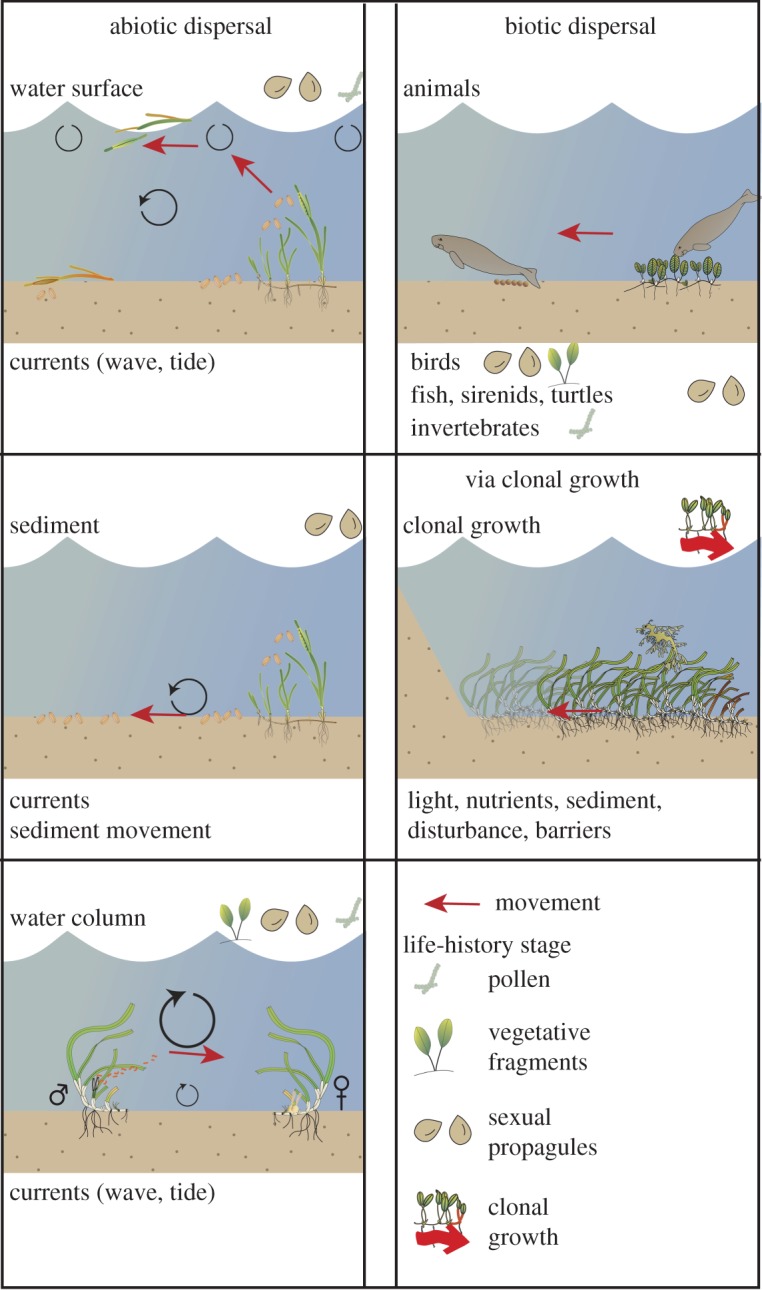
The five types of seagrass motion capacity (*Ω*), three influenced by abiotic vectors, one by biotic vectors and the final via clonal growth. For each motion capacity, the life-history stages moved, and the key attributes of the vector or environment influencing movement are identified.

We have identified five types of motion capacity for seagrasses: three associated with abiotic dispersal via water motion-on the water surface, in the water column, along the sediment; one associated with animal vectors and one via clonal growth (figure 1). For each type of motion capacity, a different set of life-history stages will be moved, each with a set of traits, that interacts with external factors to influence movement (electronic supplementary material, S1).

### (a) Surface movement

Positively, buoyant propagules and pollen are transported on the water surface. For pollen, 1 of 13 genera (*Enhalus*) is solely surface pollinated, and another three have a combination of surface and submarine pollination [37]. For reproductive propagules, seven genera have moderate to good buoyancy and six have poor buoyancy, and other reproductive structures such as rhipidia are also buoyant [38,39] (electronic supplementary material, S1). In general, the key traits for movement on the water surface are buoyancy, longevity, size, shape and timing of release. Here, the key external factors influencing movement are currents generated from tides, wind, waves, seas and swell [40]. Relative to other motion capacities, surface wind drag (windage) can significantly increase the distance that individuals move [15]. Key environmental factors that are likely to trigger the release of pollen and sexual propagules include temperature, light, aerial exposure and tides [25,29,30].

### (b) Submarine movement—water column

Neutrally buoyant pollen, sexual propagules and vegetative fragments move in the water column. Twelve of 13 genera are capable of submarine pollination, sexual propagules of six genera have poor buoyancy (electronic supplementary material, S1) so would be transported in the water column (or along the sediment) and vegetative fragments of most genera are believed to be neutrally buoyant, but few studies have actually tested this. Submarine movement is affected directly by hydrodynamics. Key traits and external factors influencing submarine water column movement are the same as those influencing surface movement. In addition, the location of release (i.e. at the plant base, within or above canopy) can have a significant influence on pollen movement [41,42] and timing of release in some genera has been associated with greater water motion (e.g. winter with high oscillatory motion for *Posidonia* and spring tides for *Thalassia* [30–32]). Landscape features such as meadow structure (density, morphology and distribution of patches and meadows in the landscape) will also greatly influence the movement, particularly of pollen [25].

### (c) Submarine movement—in and along the sediment

Only negatively buoyant sexual propagules, such as seeds will move along the sediment. Eleven seagrass genera produce seeds, which, once dehisced, are negatively buoyant (electronic supplementary material, S1). Here, motion capacity is influenced by hydrodynamics of the bottom boundary layer (water and sediment) for re-suspension or transportation processes. Seeds can be moved along the sediment surface by wave driven oscillatory flows (energetic threshold approx. 60 mPa) which are common in shallow inshore areas [15], although distances will be small (metres), particularly if seeds are covered by sediment or protected from bottom velocities by small-scale topographic features [43,44]. Once covered by sediment, movement is only likely with large, highly energetic events that mobilize seeds and the sediment as bed load transport [45]. Once again, the key traits influencing transport are buoyancy, longevity, size and shape (electronic supplementary material, S1). Compared with the previous motion capacities, movement on top of or in sediment is likely to have the shortest movement paths. However, this is dependent on longevity of the seed (electronic supplementary material, S1).

### (d) Animal vectors

Dugongs, manatees, turtles, waterfowl and fishes can move seeds through consumption and faecal deposition. Pollen, sexual propagules and fragments can also be moved attached to animals, and fragments generated through bioturbation of the sediment (e.g. shrimp) or grazing (e.g. birds). The distances that biological vectors transport seagrass material ranges from metres to 1000 km. Invertebrates facilitate pollen movement for *Thalassia* over metres [17]. Birds have moved vegetative fragments and seeds of *Ruppia* and *Zostera* over hundreds of kilometres potentially across continents, and fishes (200 m) and turtles (up to 1.5 km) have moved *Zostera* seeds significant distances [19]. Based on the movement paths of dugongs, seeds could be moved 1000s km over short timescales [20,46] overlapping with the duration that seeds pass through the digestive track (6–7 days [47]). Although biotic dispersal has been documented, the significance is poorly understood.

### (e) Via clonal growth

Seagrass genets move by growing horizontally and vertically, branching and adding new ramets onto the existing plant, a process controlled by apical dominance that influences their motion capacity. Clonal growth is the only motion capacity that is not dependent on dispersal vectors and that can be directly influenced in an ecological sense by navigation capacity. Genet's are able to grow towards nutrient rich locations [48] and gaps in the canopy [49] or away from stressful sulfide-rich patches [50]. Clonal growth occurs throughout a genets life, with seasonal variation and in response to environmental conditions such as nutrients [33], light [35] and grazing [34]. Substrate barriers can also influence this motion capacity. The movement path via clonal growth is dependent on growth rates and longevity. Clonal growth varies over two orders of magnitude across seagrass genera, from 1 cm year^−1^ (*Posidonia oceanica*) to up to 4 m year^−1^ (*Halophila* [51]; electronic supplementary material, S1) and for multiple species in at least four genera is positively correlated with age, so that older patches expand faster [52]. Genet lifespans are poorly known but have been inferred from genetic studies, with ages exceeding 2000 years for *Zostera* [53] to up to 100 000 years for *Posidonia* [54]. Based on clonal sizes, and hence the distances grown, movement more than 10 km has been estimated for species with extreme longevity [54].

## 7. Movement path case studies

**Figure 2.**
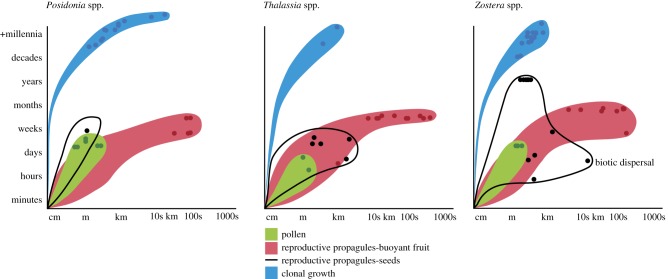
Estimated movement paths, over space and time for different seagrass life-history stages. The shape indicates the predicted footprint of movement, with coloured dots depicting actual data points (refer to the electronic supplementary material, S2).

The movement path for the lifespan of each life-history stage has different space and time footprints, that reflects the interaction between the mode of movement, traits that influence movement and a range of external factors as described above (figure 2). The space–time movement paths have been summarized for three seagrass genera, *Posidonia*, *Thalassia* and *Zostera* (figure 2) from the available data (electronic supplementary material, S2) and show the maximum measured movement for each life-history stage. There were no data on movement paths for vegetative fragments, so it was not included. Data were most extensive for sexual propagules and clonal growth, and least extensive for pollen. Where data were scarce or restricted in spatial extent, elements of these movement paths may be underestimated. However, within genera, life-history stage spanned different countries or species, giving us some confidence in the broader applicability of this analysis. A probability distribution of the distance each life-history stage moves and the success of movement (i.e. pollination, seedling establishment) would greatly increase our understanding of the significance of movement, however, this information is very limited in the seagrass literature. We present one modelled case study for *Posidonia* fruits.

### (a) Posidonia

*Posidonia* pollen is filamentous, up to 1 mm long, forming crescent shapes that reduce sinking rates. It is also released at times of high oscillatory or tidal currents, over a period of 7 days. Dispersal occurs over spatial scales of metres to hundreds of metres and timescales of tens of hours to days, pollen can be viable up to 50 h after release [37,41].

Fruits of *Posidonia* are large (approx. 24 mm length), positively buoyant and can float for days to weeks [15,55] and during this time can be driven by winds and currents distances of tens to hundreds of kilometres. Seeds are large (20 mm length), have no dormancy, are negatively buoyant and rapidly settle to the bottom, dispersing metres after release. Once settled, they are rarely resuspended, though it can occur in the first days to weeks after settlement [15]. These dispersal distances are supported by population genetic studies of *P. oceanica* [56]. Although these times and distances can be reached, many seeds are released from fruit while still attached or near to their parent plants [57], as demonstrated below.

**Figure 3.**
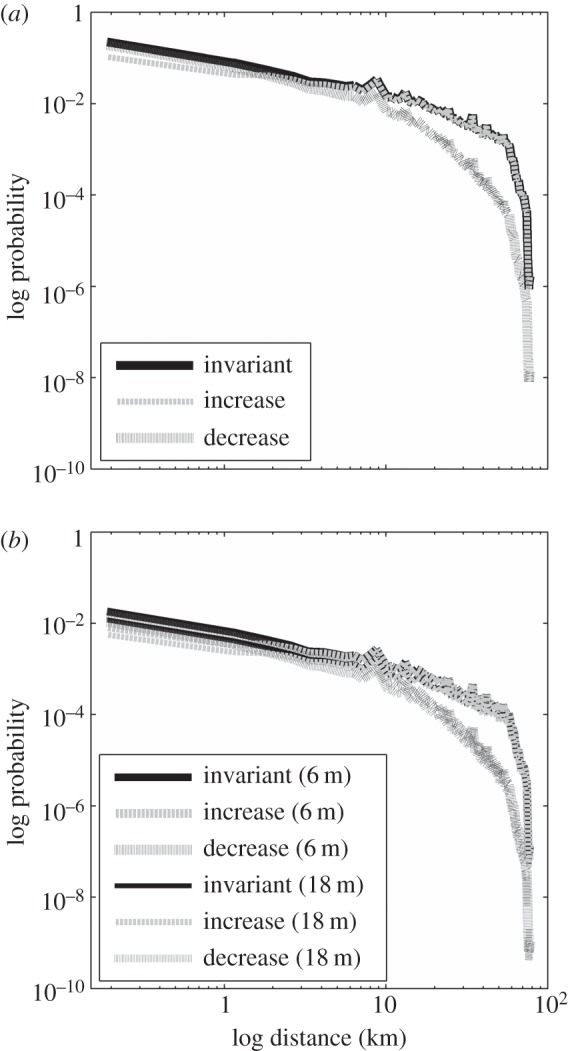
Probability distribution (proportion) of the distance (km) *Posidonia australis* (*a*) buoyant fruit and seed travel under three scenarios which vary based on the survivorship of propagules with distance from the source of release (invariant, increase or decrease with distance from source) and (*b*) seedling survival after six months and 18 months (refer to the electronic supplementary material, S3).

The distance and direction that *Posidonia australis* fruit and seeds move and settle can be approximated incorporating differential survival of seeds owing to predation or environmental conditions. The more interesting question for movement is understanding the survival of seeds with distance from parent source, essentially the success of this movement. This is very difficult to estimate as the role of density dependence or seed viability after lengthy transport times on the survival of recruiting populations is poorly understood. Here, we start this important discussion by modelling the dispersal shadow for *P. australis* using different models of post-dispersal seed survival with distance from source (no change, increase or decrease of survivorship), and estimates of seedling survival (figure 3 and electronic supplementary material, S3). We demonstrate that most seeds settle within the first few metres to kilometres of their parents; 1.2–2.4% of all seeds will travel at least 10 km, 0.4–1.5% 20 km and 0.01–0.04% will travel over 70 km (range from three model outputs). If seed survivorship declines with distance from the release location, the probability of reaching greater distances is reduced. Assuming that 100 000 seeds are released from a single source, which is a realistic assumption (G. A. Kendrick 2011, unpublished data), 1200–2400 seeds would travel 10 km, 96–192 recruits (8%) would survive for six months and 63–127 juveniles (5%) develop to 18 months. Of those that travel 20 km, around 21–79 juveniles would survive to 18 months, and those that travel 70 km, up to two would survive to 18 months. Clearly, fruit and seeds are transported long distances, irrespective of the type of seed survivorship model and have the potential to recruit in low numbers. Considering widespread seed production along 1000 s km of coastline in temperate Australia annually, contemporary movement of seeds across 10 s of km is critical for recruitment into, and persistence of, meadows across ecological timescales.

*Posidonia* species are long lived, and individuals can grow clonally over decades to millennia [54]. Growth is slow, 10s to 100s mm yr^−1^ [51] that over decades can occupy extensive areas (100s km^2^) [58,59].

### (b) Thalassia

*Thalassia* has a similar movement path to *Posidonia* for most life-history stages as illustrated by the spatial and temporal extent of the movement footprints (figure 2), and also has buoyant fruit with no seed dormancy but is estimated to be shorter lived and grows faster (electronic supplementary material, S1). The pollen movement footprint is slightly reduced compared with *Posidonia* possibly owing to pollen release at the boundary layer between the sediment and water column, rather than within or above the canopy, and the shorter viability time, 18 h [30]. Although fruits of *Thalassia* are a similar size and have a similar floating time to *Posidonia* [13,14,60,61], genetic connectivity approaches indicate the potential dispersal distances are greater, up to 720 km [13]. However, the dehiscence of fruits attached to the parent plant occurs nine times of 10, so most seeds are dispersed within metres of parent plants. By contrast, seeds can float for a limited time (less than 1 day) so they do have the potential to disperse further, and bottom speeds of 1 m d^−1^ and distances up to 13 km has been recorded [14,61]. Although *Thalassia* grows slightly faster than *Posidonia*, range 19–69 versus 2–12 cm yr^−1^ [60,62,63] (electronic supplementary material, S1), the age estimates are two orders of magnitude less (electronic supplementary material, S2), and the spatial extent of the movement path is not as long as *Posidonia*, i.e. 750 m versus 15 km.

### (c) Zostera

The shape of the *Zostera* movement footprint is least similar to the other two genera. The seed movement path is spatially longer owing to biotic dispersal and temporally longer owing to seed dormancy. In addition, the spatial extent of the clonal movement path is shorter, as the estimated age of individuals, in most examples, is less than in the other two case studies. But owing to the similar pollen viability time [64,65], the pollen movement path does not have a different shape. Pollen can move up to 15 m, but most is trapped by the leaf canopy within the first 0.5 m [66]. Seeds can be dispersed by positively buoyant, flowering branches (rhipidia), up to 100s of kilometres and can be transported for weeks to months of transport (electronic supplementary material, S1) [38,39,67,68]. Biotic vectors can disperse seeds through consumption or entanglement of reproductive material and seeds are dormant and viable for 12 months or more [69]. This explains the larger shape of the movement path for seeds compared to the other genera. Secondary dispersal of seeds occurs through resuspension of sediments during storms, but they only travel metres [43] and owing to small gas bubbles which adhere to the seedcoat, enabling the seeds to float for up to 40 min and over 200 m, although this only affects 5–13% of the seeds [70]. *Zostera* has the fastest growth rate out of the three case studies, but its maximum age [53] is a similar order of magnitude to *Thalassia*, and these two genera have similar movement paths for clonal growth.

## 8. Conclusion about movement and its consequences in seagrass

This review has addressed the various mechanisms of movement in seagrasses within a movement ecology framework, for an improved understanding of the role and consequences of movement in seagrasses over ecological and evolutionary timeframes. Life-history stages were shown to vary in their space–time movement footprint. A key conclusion drawn is that reproductive propagules and vegetative expansion via clonal growth can move over similar distances, but the timescales over which they do this range exponentially, from hours to months to centuries to millennia. Consequently, the environmental factors and key traits that interact to influence movement of these different life-history stages also operate on vastly different spatial and temporal scales. For example, interaction between traits to facilitate release of sexual propagules at a time when dispersal would be enhanced via currents is key for long-distance dispersal, whereas interactions with traits to facilitate persistence of individuals in the environment are integral for clonal expansion.

A capacity for long-distance dispersal over multiple time frames is a novel feature of the movement ecology of seagrasses. The low speciation rate in seagrasses is in marked contrast to the radiation of terrestrial angiosperms at the end of the Cretaceous [7]. A high level of genetic connectivity through movement among populations contributes a cohesive force against allopatric speciation. These conclusions also require us to reconsider the notion of a trade-off between clonality and sexual reproduction in clonal angiosperms [71], where investment in clonality can reduce investment in sexual reproduction, which is common for aquatic plants [72]. In our exploration of seagrass movement ecology, there has been little evidence of low reproductive output. Although not well studied, the evidence indicates large production and release of seeds. Dispersal of these seeds can be hundreds of kilometres within ecological timeframes, but dispersal through clonality can also be extensive over evolutionary timeframes. Extensive connectivity among seagrass populations over large distances is supported by population genetic data [56]. Movement of seagrasses over multiple timeframes has significant ecological and evolutionary consequences.

Our application of a movement ecology framework to seagrasses has identified an important transition in the life cycle between reproduction and recruitment of individuals, where movement of both pollen and seeds occurs. The greatest opportunity for long-distance movement and demographic connectivity among distant populations within contemporary timeframes is via seeds. These events will be critical for the long-term resilience of seagrass populations in the face of global threats and declines [9] via the establishment or re-establishment of plants from sexual propagules, as connectivity to established populations is the main driver of recovery of degraded seagrass habitat [73]. Yet recruitment from seeds, a measure of the success of movement, is the least studied set of life stage transitions for seagrasses. The seagrass literature is replete with single observations of seed dispersal events [56], whereas our concern is successful recruitment and establishment of seagrasses, or net dispersal of seeds, which is more ecologically relevant to the persistence and maintenance of meadows. Over smaller spatial scales, the movement of pollen is imperative for maximizing cross-pollination, yet the success of this has rarely been measured [27], in part, owing to the significant challenges posed by conducting genetic parentage assignment in dense and often clonal meadows.

Clonal growth and the external factors influencing growth has been the main focus of research in seagrasses for many years, but the interaction between ramet age, linear rhizome growth and rates of rhizome branching are less studied [74]. We have identified clonal growth as both a key motion capacity, as well as a life-history component of seagrasses, that is relevant across both evolutionary and ecological timescales, particularly for ecological resistance to disturbance. Although these are in general features of clonality, rather than a unique feature of clonal seagrasses, widespread terrestrial clones are known [75], but appear to be the exception [76]. By contrast, widespread clones appear to be more common, identified in four genera to date, and the spreading capacity greater, in seagrasses [53,54,77]. However, a common feature of both terrestrial and aquatic clonal plants is that they generally exhibit wide variation in spatial genetic structure among populations, which indicates that movement capacity from clonality is strongly influenced by local environmental factors [77,78]. Indeed, it has been long recognized that clonal growth is a movement process that responds to spatial variation in biotic and abiotic components in both terrestrial and aquatic environments [79].

## 9. Moving forward with seagrass movement ecology

Overlaying our understanding of seagrass life history within a movement ecology framework has allowed us to identify the gaps in knowledge for assessing the significance of movement in seagrasses. Only three of 13 genera had enough data available to develop space–time movement footprints, and all of these had reproductive propagules that had some floating phase and two with no seed dormancy. Further research could focus on filling these data gaps, particularly with species that have dormant seeds, and no buoyant reproductive phase such as *Halodule* and *Cymodocea*. We recommend future research should focus on understanding:

(1) the traits important for measuring and predicting the physical movement of sexual propagules and vegetative fragments;
(2) how these traits interact with external factors;
(3) measures of successful movement, such as settlement, recruitment and survival, as well as the transition between dispersing and recruiting propagules, recruitment and clonal growth, particularly for sexual and vegetative propagules;
(4) the significance of biotic vectors in moving seagrass, and the consequences of impacts on biotic vectors for movement of seagrass;
(5) the probability distribution of the different space–time movement paths to understand the significance of different motion capacities for sexual propagules, vegetative fragments and clonal growth; and
(6) the interaction between the plant, dispersal vectors and environmental state using a combination of modelling approaches (hydrodynamic, movement ecology of the biological vector and genetic connectivity), to improve our knowledge of the mechanism, significance and consequences of movement in seagrasses.

## Supplementary Material

Supplementary material
