# Supplementary material for: The movement ecology of seagrasses
Source: Proc Biol Sci. 2014 Nov 22;281(1795):20140878. doi: 10.1098/rspb.2014.0878 (PMC4213608; doi:10.1098/rspb.2014.0878)
Supplement: Supplementary material [file rspb20140878supp1.docx]

Supplementary material

S1: Seagrass genera with key traits relating to movement.

Subscripts refer to the relevant references detailed below. Pollen movement is defined by whether it moves at the surface of the water (surface) or in the water-column (submarine). Sexual propagule is the structure that seeds can be moved, as either a seed (seed), a single or group of seeds encased in a fruit (fruit), a specialised flowering stalk containing a number of fruit (inflorescence), a specialised shoot with a large bract that contains seeds (spathe), a group of connected reproductive shoots with spathes (rhipidia) or released as a partly developed seedling from viviparous species (seedlings). The release position of sexual propagules is defined in relation to the sediment (In, On, Above) or the canopy (Base, In, Above). Where the sexual propagule is released into the sediment, it is released below the canopy, so there is no release position recorded in relation to the canopy. The buoyancy of the sexual propagule is defined as Good, Moderate or Poor, where Good indicates that it is positively buoyant, floating on the water surface, Moderate that is neutrally to positively buoyant, so can float on the water surface or be transported through the water column, and Poor indicates negatively buoyant, and is not transported in or on the water but on or in the sediment. Seed bank dormancy is defined as Indistinct: No dormancy period, Viviparous: Seedlings develop on mother plant, Transient: Seeds dormant but turnover in <1 year, Persistent: Seeds dormant but remain viable for >1 year. Rhizome extension rate is the average growth rate per year for each genera, with the range identifying within that particular genus, the species minimum and maximum average growth rate.

| Genera | Pollen movement | Sexual propagule | Release position  of sexual propagule in relation to sediment | Release position of sexual propagule in relation to canopy | Buoyancy of sexual propagule | Seed bank  dormancy | Rhizome extension rate (cm/yr) |
| --- | --- | --- | --- | --- | --- | --- | --- |
| **Hydrocharitaceae** |  |  |  |  |  |  |  |
| *Enhalus* | Surface_[_[_1_](#_ENREF_1)_]_ | Fruit, Seed_[_[_2_](#_ENREF_2)_]_ | Above_[_[_3_](#_ENREF_3)_]_ | Above_[_[_1_](#_ENREF_1)_]_ | Good_[_[_3_](#_ENREF_3)_]_ | Indistinct_[_[_3_](#_ENREF_3)_]_ | 3_[_[_4_](#_ENREF_4)_]_ |
| *Thalassia* | Submarine_[_[_1_](#_ENREF_1)_]_ | Fruit, Seed_[_[_2_](#_ENREF_2)_]_ | On_[_[_3_](#_ENREF_3)_]_ | Base_[_[_1_](#_ENREF_1)_]_ | Good_[_[_3_](#_ENREF_3)_]_ | Indistinct_[_[_3_](#_ENREF_3)_]_ | 54-69_[_[_4_](#_ENREF_4)_]_ |
| *Halophila* | Submarine_[_[_1_](#_ENREF_1)_]_ | Fruit, Seed_[_[_2_](#_ENREF_2)_]_ | In - above_[_[_3_](#_ENREF_3)_]_ | In_[_[_1_](#_ENREF_1)_]_ | Moderate/Poor_[_[_3_](#_ENREF_3)_]_ | Transient/Persistent_[_[_3_](#_ENREF_3)_]_ | 356_[_[_4_](#_ENREF_4)_]_ |
| **Cymodoceaceae** |  |  |  |  |  |  |  |
| *Amphibolis* | Submarine_[_[_1_](#_ENREF_1)_]_ | Seedling_[_[_2_](#_ENREF_2)_]_ | Above_[_[_3_](#_ENREF_3)_]_ | In_[_[_1_](#_ENREF_1)_]_ | Good_[_[_3_](#_ENREF_3)_]_ | Viviparous_[_[_3_](#_ENREF_3)_]_ | 4-20_[_[_4_](#_ENREF_4)_]_ |
| *Cymodocea* | Submarine_[_[_1_](#_ENREF_1)_]_ | Fruit_[_[_2_](#_ENREF_2)_]_ | In_[_[_3_](#_ENREF_3)_]_ | - | Poor_[_[_3_](#_ENREF_3)_]_ | Persistent_[_[_3_](#_ENREF_3)_]_ | 40-210_[_[_4_](#_ENREF_4)_]_ |
| *Halodule* | Submarine_[_[_1_](#_ENREF_1)_]_ | Fruit_[_[_2_](#_ENREF_2)_]_ | In_[_[_3_](#_ENREF_3)_]_ | - | Poor_[_[_3_](#_ENREF_3)_]_ | Persistent_[_[_3_](#_ENREF_3)_]_ | 101-223_[_[_4_](#_ENREF_4)_]_ |
| *Syringodium* | Submarine_[_[_1_](#_ENREF_1)_]_ | Fruit_[_[_2_](#_ENREF_2)_]_ | Above_[_[_3_](#_ENREF_3)_]_ | In-above_[_[_1_](#_ENREF_1)_]_ | Poor_[_[_3_](#_ENREF_3)_]_ | Persistent_[_[_3_](#_ENREF_3)_]_ | 109-123_[_[_4_](#_ENREF_4)_]_ |
| *Thalassodendron* | Submarine_[_[_1_](#_ENREF_1)_]_ | Seedling_[_[_2_](#_ENREF_2)_]_ | Above_[_[_3_](#_ENREF_3)_]_ | In_[_[_1_](#_ENREF_1)_]_ | Good_[_[_3_](#_ENREF_3)_]_ | Viviparous_[_[_3_](#_ENREF_3)_]_ | 3-16_[_[_4_](#_ENREF_4)_]_ |
| **Posidoniaceae** |  |  |  |  |  |  |  |
| *Posidonia* | Submarine_[_[_1_](#_ENREF_1)_]_ | Inflorescence, Fruit, Seed_[_[_2_](#_ENREF_2)_]_ | Above_[_[_3_](#_ENREF_3)_]_ | In-above_[_[_1_](#_ENREF_1)_]_ | Good_[_[_3_](#_ENREF_3)_]_ | Indistinct_[_[_3_](#_ENREF_3)_]_ | 2-12_[_[_4_](#_ENREF_4)_]_ |
| **Zosteraceae** |  |  |  |  |  |  |  |
| *Phyllospadix* | Surface & submarine_[_[_1_](#_ENREF_1)_]_ | Rhipidia, Spathe, Seed_[_[_2_](#_ENREF_2)_]_ | Above_[_[_3_](#_ENREF_3)_]_ | In-above_[_[_1_](#_ENREF_1)_,_ [_5_](#_ENREF_5)_]_ | Moderate_[_[_3_](#_ENREF_3)_]_ | Transient_[_[_3_](#_ENREF_3)_]_ | 17-26_[_[_4_](#_ENREF_4)_]_ |
| *Zostera* | Surface & submarine_[_[_1_](#_ENREF_1)_]_ | Rhipidia, Spathe, Seed_[_[_2_](#_ENREF_2)_]_ | Above_[_[_3_](#_ENREF_3)_]_ | In-above_[_[_1_](#_ENREF_1)_]_ | Good/Moderate_[_[_3_](#_ENREF_3)_]_ | Transient_[_[_3_](#_ENREF_3)_]_ | 26-68_[_[_4_](#_ENREF_4)_]_ |
| **Ruppiaceae** |  |  |  |  |  |  |  |
| *Ruppia* | Surface & submarine_[_[_6_](#_ENREF_6)_]_ | Seed_[_[_6_](#_ENREF_6)_]_ | Above_[_[_6_](#_ENREF_6)_]_ | In-above_[_[_6_](#_ENREF_6)_]_ | Poor_[_[_7_](#_ENREF_7)_]_ | Persistent_[_[_7_](#_ENREF_7)_]_ | nd |
| **Potamogetonaceae** |  |  |  |  |  |  |  |
| *Lepilaena* | Submarine_[_[_2_](#_ENREF_2)_]_ | Fruit_[_[_2_](#_ENREF_2)_]_ | Above_[_[_2_](#_ENREF_2)_]_ | In_[_[_2_](#_ENREF_2)_]_ | Poor_[_[_2_](#_ENREF_2)_]_ | Persistent_[_[_8_](#_ENREF_8)_]_ | nd |
|  |  |  |  |  |  |  |  |

References

1. Ackerman J.D. 2006 Sexual reproduction of seagrasses: Pollination in the marine context. In *Seagrasses: Biology, Ecology and Conservation* (eds. Larkum A.W.D., Orth R.J., Duarte C.M.), pp. 89-109. Dordrecht, Springer.

2. Jacobs S.W.L., McColl K.A. 2011 Zannichelliaceae. In *FLora of Australia Volume 39 Alismatales to Arales.* Melbourne, Australia, ABRS/CSIRO.

3. Orth R.J., Harwell M.C., Inglis G.J. 2006 Ecology of seagrass seeds and dispersal strategies. In *Seagrasses: Biology, Ecology and Conservation* (eds. Larkum A.W.D., Orth R.J., Duarte C.M.), pp. 111-133. Dordrecht, The Netherlands, Springer.

4. Duarte C.M., Fourqurean J.W., Krause-Jensen D., Olesen B. 2006 Dynamics of seagrass stability and change. In *Seagrasses: biology, ecology and conservation* (eds. Larkum A.W.D., Orth R.J., Duarte C.M.), pp. 271-294. Dordrecht, The Netherlands, Springer.

5. Cox P.A., Laushman R.H., Ruckelshaus M.H. 1992 Surface and submarine pollination in the seagrass *Zostera marina* L. *Bot. J. Linn. Soc.* **109**(2), 281-291. (doi:10.1111/j.1095-8339.1992.tb00272.x).

6. Jacobs S.W.L., Brock M.A. 2011 Ruppiaceae In *Flora of Australia Volume 39, Alismatales to Arales.* Melbourne, Autralia, ABRS/CSIRO.

7. Ailstock S.M., Shafer D.J., Magoun D.A. 2010 Protocols for use of *Potamogeton perfoliatus* and *Ruppia maritima* seeds in large‐scale restoration. *Restor. Ecol.* **18**(4), 560-573. (doi:10.1111/j.1526-100X.2010.00696.x).

8. Porter J.L., Kingsford R.T., Brock M.A. 2007 Seed banks in arid wetlands with contrasting flooding, salinity and turbidity regimes. *Plant Ecol.* **188**(2), 215-234. (doi:10.1007/s11258-006-9158-8).

S2: Data supporting the space-time movement paths of the seagrass genera case-studies, *Thalassia*, *Posidonia* and *Zostera* illustrated in Figure 2.

Each line in the table below is an example of space and time movement and is depicted as a dot in Figure 2. The table is arranged by genera (*Thalassia*, *Posidonia* and *Zostera*) and dispersal unit (P-pollen, Sexual propagules: F-buoyant fruit; R-buoyant rhipidia; S- non-buoyant seed, and C-clonal growth). For each example the species, reference from which the data was extracted and country of study is identified. In some cases, more than one reference was used to extract the data. This was because in these cases the same data was reported on, or referred to, but different variables (i.e. dispersal distance, speed or time) were reported in different papers. The space and time movement footprint for each example was calculated from a combination of the quoted dispersal distance (average or maximum), dispersal speed (maximum) and dispersal time (maximum). The dispersal time is analogous to the viability time of pollen or fruit and seeds and the life span or age of a genet. The dispersal distance was recorded as either average or maximum, or both. As there was no consistent approach in the literature we examined, and in most cases it was not possible to calculate either the maximum or the average dispersal distance, maximum and average dispersal distance were included in Figure 2. Where more than one dispersal speed or time was recorded, only the maximum was included. In 15 of the 81 examples both average and maximum dispersal distance were estimated and these were both included in Figure 3. For calculating dispersal time or speed, in some examples assumptions were made (noted by _a_). These assumptions are identified in the column next to dispersal time and if this assumption was derived from a different reference, this is noted. Age of genets was inferred using the clone size and estimated growth rate. Clones sizes were generally associated with a particular population, so where applicable populations were listed separately. For some references there are multiple entries in the table below as more than one species (e.g. reference 9, 14) and/or dispersal unit or life-history stage were described (e.g. reference 4, 5, 10). Also in some cases, there are multiple entries where dispersal estimates were given for different regions or ocean basins, and these are reported separately as the characteristics of the location, particularly current speed and direction, can greatly influence the distance moved (e.g. reference 4, 5, 10). Finally, for calculating clonal growth movement paths, it was necessary to estimate the age of the clone or genet. As clone size and age can vary greatly with location, due to local processes, where multiple populations were identified with different clone sizes, and hence estimated ages, these were reported separately (e.g. reference 33, 34).

| **Taxa** | **Dispersal Unit** | **Dispersal Distance** | **Dispersal Distance** | **Dispersal**  **Speed** | **Dispersal**  **time** | **Assumptions**  **[references in brackets]** | **Country** | **Reference** |
| --- | --- | --- | --- | --- | --- | --- | --- | --- |
|  |  | Average | Maximum | Maximum | Maximum |  |  |  |
| ***Thalassia*** |  |  |  |  |  |  |  |  |
| *testudinum* | P | 1.5 m |  |  | 5 h_a_ | based on tidal cycle (4-6 h) | U.S. Virgin Islands | 1 |
| *testudinum* | P | 1 m |  | 25 cm s^-1^ | 18 h_a_ | viable for 18 h [2] | Mexico | 3 |
| *testudinum* | F |  | 1022 m | 676m h^-1^ | 138 min |  | Mexico | 4 |
| *testudinum* | F | 10 km | 95 km |  | 10 d |  | Mexico | 4 |
| *testudinum* | F |  | 720 km | 3 km h^-1^ | 10 d |  | Mexico | 4 |
| *testudinum* | F | 7.7 km | 350 km |  | 10 d |  | Mexico | 4 |
| *hemprichii* | F | 23.4 km | 73.5 km | 0.43 km h^-1^ | 11 d |  | Philippines | 5 |
| *hemprichii* | F |  | 300 km | 1.8 km h^-1^ | 11 d |  | Philippines | 5 |
| *testudinum* | F |  | 15 km | 1.5 km d^-1^ | 10 d |  | USA | 7 |
| *testudinum* | F |  | 100 km |  | 10 d |  | Mexico | Pers. comm. KJ van Dijk |
| *testudinum* | S | 1-2 m |  |  | 3 d |  | Mexico | 4, 6 |
| *hemprichii* | S |  | > 2 m | 10.3 cm h^-1^ | 2 d |  | Philippines | 5 |
| *hemprichii* | S |  | 1.8 km | 0.52 km h^-1^ | 3.5 h |  | Philippines | 5 |
| *hemprichii* | S |  | < 100 m |  | 2 d |  | Philippines | 5 |
| *testudinum* | S |  | 3 km | 1.5 km d^-1^ | 3 d |  | USA | 7 |
| *testudinum* | C |  | 230 m | 19 cm y^-1^ | 595 y |  | Mexico | 8 |
| *testudinum* | C |  | 750 m | 19 cm y^-1^ | 2000 y |  | Bermuda | Pers. comm. KJ van Dijk |
|  |  |  |  |  |  |  |  |  |

| **Taxa** | **Dispersal Unit** | **Dispersal Distance** | **Dispersal Distance** | **Dispersal**  **Speed** | **Dispersal**  **time** | **Assumptions** | **Country** | **Reference** |
| --- | --- | --- | --- | --- | --- | --- | --- | --- |
|  |  | Average | Maximum | Maximum | Maximum |  |  |  |
| ***Posidonia*** |  |  |  |  |  |  |  |  |
| *australis* | P | 30 cm | 1.2 m |  | 50 h |  | Australia | 9 |
| *sinuosa* | P | 20 cm | 1.2 m |  | 50 h |  | Australia | 9 |
| *australis* | P | 30.8 m | 178.2 m |  | 50 h |  | Australia | 10 |
| *australis* | P | 26.8 m | 74.5 m |  | 50 h |  | Australia | 10 |
| *oceanica* | F |  | 80 km |  | 9-14 d |  | Italy | 11, 12 |
| *oceanica* | F |  | 100 km |  | 9-14 d_a_ | [12] | Mediterranean var. | 13, 14 |
| *australis* | F |  | 100 km |  | 5 d |  | Australia | 14, Pers comm L. Ruiz-Montoya |
| *australis* | F |  | 55 km | 0.23 km h^-1^ | 5 d |  | Australia | 14 |
| *australis* | F |  | 76 km |  | 5 d |  | Australia | 15 |
| *oceanica* | S | 40 m |  |  |  |  | Italy | 16 |
| *australis* | S |  | 10 m |  | 5 mth |  | Australia | Pers. comm. J. Statton |
| *australis* | C | 12.8 m | 41 m |  | 820 y_a_ | [17] | Australia | 10 |
| *oceanica* | C |  | 80 m | 4 cm y^-1^ | < 600 y |  | Greece | 13, 20, 21 |
| *oceanica* | C |  | 140 m |  | 650-4500 y | Size based on presented map | Italy | 16 |
| *oceanica* | C | 70 m |  |  | 233-1750 y_a_ | Age inferred from growth measures | Italy | 16 |
| *oceanica* | C | 4 m |  |  | 30-200 y |  | Italy | 16 |
| *australis* | C |  | 12 m | 2.5 cm y^-1^ | 480 y |  | Australia | 17 |
| *australis* | C |  | 60 m |  | 1200y_a_ | [17] | Australia | 18 |
| *oceanica* | C |  | 1000 m | 1 cm y^-1^ | 100000 y |  | Slovenia, Italy | 19 |
| *oceanica* | C |  | 1 km | 4 cm y^-1^ | 12500 y |  | Italy | 20 |
| *oceanica* | C |  | 1 km | 4 cm y^-1^ | 12500 y |  | Cyprus | 20 |
| *oceanica* | C |  | 1 km | 4 cm y^-1^ | 12500 y |  | Spain | 20 |
| *oceanica* | C |  | 15 km | 4 cm y^-1^ | 200000 y |  | Spain | 20 |
| *oceanica* | C |  | 7 m | 4 cm y^-1^ | 80000 y |  | Spain | 20 |
|  |  |  |  |  |  |  |  |  |

| **Taxa** | **Dispersal Unit** | **Dispersal Distance** | **Dispersal Distance** | **Dispersal**  **Speed** | **Dispersal**  **time** | **Assumptions** | **Country** | **Reference** |
| --- | --- | --- | --- | --- | --- | --- | --- | --- |
|  |  | Average | Maximum | Maximum | Maximum |  |  |  |
| ***Zostera*** |  |  |  |  |  |  |  |  |
| *marina* | P | 1.1 m | 15 m |  | 48 h_a_ | [22] | USA | 23 |
| *marina* | P |  | 2-4 m |  | 48 h_a_ | [22] | Germany | 31 |
| *marina* | R |  | 34 km | 3.8 km h^-1^ | 3 w |  | USA | 24 |
| *marina* | R |  | 7.3 km |  | 26 d_a_ | [27] | USA | 25 |
| *marina* | R |  | 108 km | 3.8 km h^-1^ | 3 w |  | USA | 25 |
| *marina* | R | few km | 150 km |  | 26 d |  | Sweden | 27 |
| *marina* | R |  | 54 km |  | 26 d_a_ | [25] | Germany | 28 |
| *marina* | R |  | few km | 8m s^-1^ | 26 d_a_ | [27] | Germany | 28 |
| *marina* | R |  | 100 km |  | 26 d_a_ | [27] | North Europe | 33 |
| *marina* | R |  | 150 km |  | 26 d_a_ | [27] | North Europe | 33 |
| *noltii* | R |  | 100-150 km |  | 26 d_a_ | [27] | Europe | 34 |
| *noltii* | R |  | 100 km |  | 26 d_a_ | [27] | Portugal, Spain | 35, 34 |
| *marina* | R |  | 200 km | 100 cm s^-1^ | 2.3 d |  | Mexico | 36 |
| *marina* | R |  | 150 km |  | 26 d_a_ | [27] | Netherlands, Germany, Denmark | 37 |
| *marina* | R |  | < 25 km |  | 26 d_a_ | [27] | USA | 38 |
| *marina* | S | 1.27 m | < 50 m |  | 1 y_a_ | [25] | USA | 23 |
| *marina* | S | < 5 m | 14 m |  | 1 y |  | USA | 25 |
| *marina* | S |  | 1.3 m |  | 1 y_a_ | [25] | Great Britain | 26 |
| *marina* | S |  | 200 m |  | > 40 min |  | USA | 29 |
| *marina* | S |  | 200 m |  | 15-20 h |  | USA | 30 |
| *marina* | S |  | 60 m |  | 7-> 10 h |  | USA | 30 |
| *marina* | S |  | 1500 m |  | 24-144 h |  | USA | 30 |
| *marina* | S |  | 19.5 km |  | 2-5 h |  | USA | 30 |
| *marina* | S |  | 27 m |  | 1 y_a_ | [25] | Germany | 32 |
| *marina* | C |  | 160 m | 10 cm y^-1^ | 1600 y |  | Germany | 32 |
| *marina* | C | 2-4 m | > 50 m | 10 cm y^-1^_a_ | 250 y | [32] | Denmark | 33 |
| *marina* | C |  | 10 m | 10 cm y^-1^_a_ | 50 y | [32] | Portugal | 33 |
| *marina* | C |  | > 75 m | 10 cm y^-1^_a_ | 375 y | [32] | Ukraine | 33 |
| *marina* | C |  | > 20 m | 10 cm y^-1^_a_ | 100 y | [32] | USA | 33 |
| *marina* | C |  | > 50 m | 10 cm y^-1^_a_ | 250 y | [32] | USA | 33 |
| *marina* | C |  | 50 m | 26 cm y^-1^ | 96-192 y |  | Ukraine | 34 |
| *noltii* | C | 3 m^2^ | 6 m | 68 cm y^-1^ | 9 y |  | Germany | 34 |
| *noltii* | C |  | 50 m | 68 cm y^-1^ | 34-69 y |  | Ukraine | 34 |
| *noltii* | C |  | 46 m | 68 cm y^-1^ | 29-59 y |  | Ukraine | 34 |
| *noltii* | C |  | 30 m | 68 cm y^-1^ | 21-42 y |  | Mauretania | 34 |
| *marina* | C |  | 17 m | 10 cm y^-1^ | 67 y |  | Germany | 39 |
| *marina* | C | 7 m |  | 10 cm y^-1^_a_ | 35 y | [32] | Germany | 40 |
| *marina* | C |  | 16 m | 10 cm y^-1^_a_ | 80 y | [32] | Germany | 41 |
|  |  |  |  |  |  |  |  |  |

References

1. Cox P.A., Tomlinson P.B. 1988 Pollination ecology of a seagrass, *Thalassia testudinum* (hydrocharitaceae), in St. Croix. *Am. J. Bot.* **75**, 958-965. (doi:10.2307/2443761).

2. Larkum A.W.D., McComb A.J., Shepherd S.A. 1989 *Biology of seagrasses: a treatise of the biology of seagrasses with special reference to the Australian region*. New York, Elselvier.

3. van Tussenbroek B.I., Monroy-Velazquez V.L., Solis-Weiss V. 2012 Meso-fauna foraging on seagrass pollen may serve in marine zoophilous pollination. *Mar. Ecol. Prog. Ser.* **469**, 1-6. (doi:10.3354/meps10072).

4. van Dijk J.K., van Tussenbroek B.I., Jiménez D., K., Márquez G., G. J., Ouborg J. 2009 High levels of gene flow and low population genetic structure related to high dispersal potential of a tropical marine angiosperm. *Mar. Ecol. Prog. Ser.* **390**, 67-77. (doi:10.3354/meps08190).

5. Lacap C.D.A., Vermaat J.E., Rollon R.N., Nacorda H.M. 2002 Propagule dispersal of the SE Asain seagrasses *Enhalus acoroides* and *Thalassia hemprichii*. *Mar. Ecol. Prog. Ser.* **235**, 75-80. (doi:10.3354/meps235075).

6. Jiménez-Durán K. 2004 Desarrollo y dispersión de frutos y semillas e *Thalassia testudinum* Banks ex König (Hydrocharitaceae), Masters thesis. Facultad de Ciencias, Universidad Nacional Autónoma de México (with English abstract).

7. Kaldy J.E., Dunton K.H. 1999 Ontogenetic photosynthetic changes, dispersal and survival of *Thalassia testudinum* (turtle grass) seedlings in a sub-tropical lagoon. *J. Exp. Mar. Biol. Ecol.* **240**(2), 193-212. (doi:10.1016/S0022-0981(99)00058-1).

8. van Dijk J.K., van Tussenbroek B.I. 2010 Clonal diversity and structure related to habitat of the marine angiosperm *Thalassia testudinum* along the Atlantic coast of Mexico. *Aquat. Bot.* **92**, 63 - 69.

9. Smith N.M., Walker D.I. 2002 Canopy structure and pollination biology of the seagrasses *Posidonia australis* and *P. sinuosa* (Posidoneaceae). *Aquat. Bot.* **74**(1), 57-70. (doi:10.1016/S0304-3770(02)00047-5).

10. Sinclair E.A., Gecan I., Krauss S.L., Kendrick G.A. 2014 Against the odds: complete outcrossing in a monoecious clonal seagrass *Posidonia australis* (Posidoniaceae). *Ann Bot*. (doi:10.1093/aob/mcu048).

11. Aliani S., Gasparini G., Micheli C., Molcard A., Peirano A. 2006 Can southern meadows of the Mediterranean seagrass *Posidonia oceanica* (L.) Delile supply northern ones? A multidisciplinary approach in the Ligurian Sea. *Biologia Marina Mediterranea* **13**, 14-18.

12. Micheli C., Spinosa F., Aliani S., Gasparini G.P., Molcard A., Peirano A. 2010 Genetic input by *Posidonia oceanica* (L.) Delilie fruits dispersed by currents in the Ligurian Sea. *Plant Biosyst.* **144**(2), 333-339. (doi:10.1080/11263501003764798).

13. Arnaud‐Haond S., Migliaccio M., Diaz‐Almela E., Teixeira S., Van De Vliet M.S., Alberto F., Procaccini G., Duarte C.M., Serrao E.A. 2007 Vicariance patterns in the Mediterranean Sea: east–west cleavage and low dispersal in the endemic seagrass *Posidonia oceanica*. *J. Biogeogr.* **34**(6), 963-976. (doi:10.1111/j.1365-2699.2006.01671.x).

14. Kendrick G.A., Waycott M., Carruthers T.J.B., Cambridge M.L., Hovey R., Krauss S.L., Lavery P.S., Les D.H., Lowe R.J., Vidal O.M.I., et al. 2012 The central role of dispersal in the maintenance and persistence of seagrass populations. *Bioscience* **62**(1), 56-65. (doi:10.1525/bio.2012.62.1.10).

15. Ruiz-Montoya L., Lowe R.J., Van Niel K.P., Kendrick G.A. 2012 The role of hydrodynamics on seed dispersal in seagrasses. *Limnol. Oceanogr.* **57**(5), 1257-1265. (doi:10.4319/lo.2012.57.5.1257).

16. Migliaccio M., De Martino F., Silvestre F., Procaccini G. 2005 Meadow-scale genetic structure in *Posidonia oceanica*. *Mar. Ecol. Prog. Ser.* **304**, 55-65. (doi:10.3354/meps304055).

17. Waycott M. 1995 Assessment of genetic variation and clonality in the seagrass *Posidonia australis* using RAPD and allozyme analysis. *Marine ecology progress series. Oldendorf* **116**(1), 289-295. (doi:10.3354/meps116289).

18. Evans S., Sinclair E., Poore A.B., Steinberg P., Kendrick G., Vergés A. 2014 Genetic diversity in threatened *Posidonia australis* seagrass meadows. *Conserv Genet* **15**(3), 717-728. (doi:10.1007/s10592-014-0573-4).

19. Ruggiero M.V., Turk R., Procaccini G. 2002 Genetic identity and homozygosity in North-Adriatic populations of *Posidonia oceanica*: An ancient, post-glacial clone? *Conserv. Genet.* **3**(1), 69-72. (doi:10.1023/A:1014207122382).

20. Arnaud-Haond S., Duarte C.M., Diaz-Almela E., Marba N., Sintes T., Serrao E.A. 2012 Implications of Extreme Life Span in Clonal Organisms: Millenary Clones in Meadows of the Threatened Seagrass Posidonia oceanica. *Plos One* **7**(2). (doi:10.1371/journal.pone.0030454).

21. Diaz-Almela E., Marba N., Duarte C.M. 2007 Consequences of Mediterranean warming events in seagrass (*Posidonia oceanica*) flowering records. *Global Change Biology* **13**(1), 224-235. (doi:10.1111/j.1365-2486.2006.01260.x).

22. De Cock A.W.A.M. 1980 Flowering, pollination and fruiting in *Zostera marina* L. *Aquat. Bot.* **9**(0), 201-220. (doi:10.1016/0304-3770(80)90023-6).

23. Ruckelshaus M.H. 1996 Estimation of genetic neighbourhood parameters from pollen and seed dispersal in the marine angiosperm *Zostera marina* L. *Evolution* **50**, 856-864. (doi:10.2307/2410857).

24. Harwell M.C., Orth R.J. 2002 Long-distance dispersal potential in a marine macrophyte. *Ecology* **83**, 3319-3330. (doi:10.1890/0012-9658(2002)083[3319:LDDPIA]2.0.CO;2).

25. Orth R.J., Luckenbach M., Moore K.A. 1994 Seed dispersal in a marine macrophyte: implications for colonization and restoration. *Ecology* **75**, 1927-1939. (doi:10.2307/1941597).

26. Tutin T. 1938 The autecology of *Zostera marina* in relation to its wasting disease. *New Phytol.* **37**(1), 50-71. (doi:10.1111/j.1469-8137.1938.tb06926.x).

27. Källström B., Nyqvist A., Åberg P., Bodin M., André C. 2008 Seed rafting as a dispersal strategy for eelgrass (*Zostera marina*). *Aquat. Bot.* **88**, 148-153. (doi:10.1016/j.aquabot.2007.09.005).

28. Reusch T.B. 2002 Microsatellites reveal high population connectivity in eelgrass (*Zostera marina*) in two contrasting coastal areas. *Limnol. Oceanogr.* **47**(1), 78-85.

29. Churchill A.C., Nieves G., Brenowitz A.H. 1985 Flotation and dispersal of eelgrass seeds by gas bubbles. *Estuaries* **8**(4), 352-354. (doi:10.2307/1351872).

30. Sumoski S.E., Orth R.J. 2012 Biotic dispersal in eelgrass *Zostera marina*. *Mar. Ecol. Prog. Ser.* **471**, 1-10. (doi:10.3354/meps10145).

31. Hämmerli A., Reusch T. 2003 Inbreeding depression influences genet size distribution in a marine angiosperm. *Mol. Ecol.* **12**(3), 619-629. (doi:10.1046/j.1365-294X.2003.01766.x).

32. Reusch T.B.H., Borström C., Stam W.T., Olsen J.L. 1999 An ancient eelgrass clone in the Baltic. *Mar. Ecol. Prog. Ser.* **183**, 301-304. (doi:10.3354/meps183301).

33. Olsen J.L., Stam W.T., Coyer J.A., Reusch T.B.H., Billingham M.R., Boström C., Calvert E., Christie H., Granger S., La Lumiäre R., et al. 2004 North Atlantic phylogeography and large-scale population differentiation of the seagrass *Zostera marina* L. *Mol. Ecol.* **13** 1923-1941 (doi:10.1111/j.1365-294X.2004.02205.x).

34. Coyer J., Diekmann O., Serrao E., Procaccini G., Milchakova N., Pearson G., Stam W., Olsen J. 2004 Population genetics of dwarf eelgrass *Zostera noltii* throughout its biogeographic range. *Mar. Ecol. Prog. Ser.* **281**, 51-62. (doi:10.3354/meps281051).

35. Diekmann O.E., Coyer J.A., Ferreira J., Olsen J.L., Stam W.T., Pearson G.A., Serrão E.A. 2005 Population genetics of *Zostera noltii* along the west Iberian coast: consequences of small population size, habitat discontinuity and near-shore currents. *Mar. Ecol. Prog. Ser.* **290**, 89-96. (doi:10.3354/meps290089).

36. Muniz-Salazar R., Talbot S.L., Sage G.K., Ward D.H., Cabello‐Pasini A. 2005 Population genetic structure of annual and perennial populations of *Zostera marina* L. along the Pacific coast of Baja California and the Gulf of California. *Mol. Ecol.* **14**(3), 711-722. (doi:10.1111/j.1365-294X.2005.02454.x).

37. Ferber S., Stam W.T., Olsen J.L. 2008 Genetic diversity and connectivity remain high in eelgrass *Zostera marina* populations in the Wadden Sea, despite major impacts. *Mar. Ecol. Prog. Ser.* **372**, 87-96. (doi:10.3354/meps07705).

38. Kamel S.J., Hughes A., Grosberg R.K., Stachowicz J.J. 2011 Fine-scale genetic structure and relatedness in the eelgrass *Zostera marina*. *Mar. Ecol. Prog. Ser.* **447**, 127-137. (doi:10.3354/meps09447).

39. Reusch T.B.H., Stam W.T., Olsen J.L. 1998 Size and estimate age of genets in eelgrass, *Zostera marina*, assessed with microsatellite markers. *Marine Biology* **133**, 519-525.

40. Reusch T.B.H., Hukriede W., Stam W.T., Olsen J.L. 1999 Differentiating between clonal growth and limited gene flow using spatial autocorrelation of microsatellites. *Heredity* **83**, 120-126.

41. Hämmerli A., Reusch T.B.H. 2003 Genetic neibourhood of clone structures in eelgrass meadows quantified by spatial autocorrelation of microsatellite markers. *Heredity* **91**, 448-455.

S3: Methods to calculate the probability distance function for fruit and seed travel, and survival after six months and 18 months for *Posidonia australis*.

There were three steps to calculate the probability distance function for fruit and seed travel (Figure 3a), and seedling survival after six months and 18 months (Figure 3b) for *Posidonia australis*. Firstly, generation of the probability distribution of fruit and seed travel distance, secondly, generation of the probability distribution which takes into account post-dispersal survivorship as a function of distance from the parent plant, and thirdly, survivorship of established seedlings.

1. Probability distribution of fruit and seed travel

The probability distribution of fruit and seed travel was derived from the probability density function for dehiscence described by [[1](#_ENREF_1)] using 150 000 modelled fruit released from 14 locations in Cockburn Sound which was coupled to a 3D hydrodynamic model over a 150 by ~50 km domain (unpublished). The position of each fruit was recorded every two hours and the distance travelled from the release point calculated, the specific age of each fruit corresponded to a probability of dehiscence or seed presence at that distance. The data from all locations was then grouped according to distance travelled at 1km intervals to obtain the total probability for each 1km interval (Figure 3a dark line - Invariant).

2. Post-dispersal survivorship as a function of distance from the parent plant

It is unlikely that there is a constant rate of post-dispersal survivorship with distance from source. In fact [4] has developed a model, which shows different patterns of survivorship from the release location depending on seed production, predation pressure and predator’s habits. This variation in survivorship of seeds has been attributed to variation in the predation rate with distance from source. For example, in the Hubell model predation is greater at the release location compared to further from the source, so survivorship is reduced here. This has been demonstrated in *P. australis* seagrass meadows [5,6] where there was a greater rate of predation within the seagrass bed (up to 53%) where seeds are released compared to further from the meadow in bare areas where only 3% of seeds were consumed. A different relationship is proposed in the McCanny model which predicts that under conditions of very high seed densities near the parents, predation reaches a satiation state which allows for some survival close to the parents, while there is loss further from the release location, so there is more survivorship closer to the release location. If the environmental conditions for seedling survival are less conducive outside the meadow due to environmental or physical conditions, then this situation is possible. Greater mortality of seedlings outside of meadows has been reported in other seagrass species, but not *P. australis* and this has been related to the environment (e.g. Rivers et al 2011).

To demonstrate how the probability distribution of fruit and seed travel would vary under different models of seed survivorship with distance from source, post dispersal survivorship of the seeds was included as a function of distance from the parent plant. The first probability distribution (1. As described above) was modified, by subtracting the loss due to differential survivorship with distance from source using two different seed survivorship functions.

Increase: Survivorship increases with distance from source (Hubbell Model)

A power function was fitted to the Orth et al data [5,6], distance from meadow or source and predation rates. This was then applied to the first probability distribution to create a new probability distribution of fruit and seed travel (see Increase model Figure 3a).

Decrease: Survivorship decreases with distance from source (similar to McCanny model)

An exponential function of survivorship was fitted to the dispersal kernel with greater survivorship close to the source (see Decrease model Figure 3a).

3. Survivorship of established seedlings

The probability distribution of recruitment of seeds with distance from source was generated by applying survival rates over the first six months (3b) based on the value of 8% survival [[3](#_ENREF_3)] and up to 18 months (3b) based on the value of 66% survival [[2](#_ENREF_2)]. The assumptions here are that the seedling mortality after 6 months [[3](#_ENREF_3)] and after 18 months [[2](#_ENREF_2)] can be generalized to all *Posidonia australis* meadows. This is a limitation, as we may expect differential rates of survivorship in different meadows. However, there is no data to our knowledge to predict this. So we may be over or underestimating the survivorship of established seedlings.

A summary of these three model outputs is given below, highlighting the number of surviving seeds (post-dispersal) or seedlings (6 and 18 months post-establishment) at different distances from the source location with a release of 100 000 seeds.

| Distance travelled  (km) | Number surviving seeds | Number surviving seedlings at 6 mo | Number surviving seedlings at 18 mo |
| --- | --- | --- | --- |
| Scenario 1: Invariant | | | |
| 9-10 | 2400 | 192 | 127 |
| 19-20 | 1500 | 120 | 79 |
| >70 | 44 | 4 | 2 |
| Scenario 2: Seed survivorship increasing with distance from source | | | |
| 9-10 | 2300 | 184 | 121 |
| 19-20 | 1500 | 120 | 79 |
| >70 | 42 | 3 | 2 |
| Scenario 3: Seed survivorship decreasing with distance from source | | | |
| 9-10 | 1200 | 96 | 63 |
| 19-20 | 400 | 32 | 21 |
| >70 | 1 | 0 | 0 |
|  |  |  |  |

**References:**

1. Ruiz-Montoya L., Lowe R.J., Van Niel K.P., Kendrick G.A. 2012 The role of hydrodynamics on seed dispersal in seagrasses. *Limnol. Oceanogr.* **57**(5), 1257-1265. (doi:10.4319/lo.2012.57.5.1257).

2. Kirkman H. 1998 Pilot experiments on planting seedlings and small seagrass propagules in Western Australia. *Mar. Pollut. Bull.* **37**(8), 460-467. (doi:10.1016/S0025-326X(99)00146-0).

3. Rivers D.O. 2011 Gap dynamics and the role of seedling recruitment in maintaining seagrass diversity in seagrass meadows. PhD Thesis, The University of Western Australia. pp. 1-82.

4. Nathan, R. and Casagrandi R. 2004. A simple mechanistic model of seed dispersal, predation and plant establishment: Janzen-Connell and beyond. *Journal of Ecology*. 92(5): p. 733-746

5. Orth R.J., Heck K.L., Tunbridge D.J. 2002, Predation on seeds of the seagrass Posidonia australis in Western Australia. *Marine Ecology-Progress Series*. 244: p. 81-88.

6. Orth, R.J., Kendrick G.A., Marion S.R. 2006 Predation on *Posidonia australis* seeds in seagrass habitats of Rottnest Island, Western Australia: patterns and predators. *Marine Ecology Progress Series*. 313
